# Simulated Injuries of the Sheep Ossicular Chain: Correlations With Their Radiological Images

**DOI:** 10.7759/cureus.60419

**Published:** 2024-05-16

**Authors:** Aiste Giniunaite, Alina Barkauskiene, Rokas Abaturovas, Saulius Rocka, Irina Arechvo

**Affiliations:** 1 Department of Neurosurgery, Klinikum Aschaffenburg, Aschaffenburg, DEU; 2 Center of Radiology and Nuclear Medicine, Vilnius University Hospital Santaros Klinikos, Vilnius, LTU; 3 Department of Otorhinolaryngology, Faculty of Medicine, Medical Academy, Vilnius University, Vilnius, LTU; 4 Center of Neurosurgery, Vilnius University Hospital Santaros Klinikos, Vilnius, LTU; 5 Department of Otorhinolaryngology, Republican Vilnius University Hospital, Vilnius, LTU

**Keywords:** middle ear trauma, ossicular chain visualization techniques, sheep temporal bone model, radiological and surgical correlations, ossicular injuries

## Abstract

Hypothesis

We hypothesized that a sheep temporal bone would be a suitable model to study correlations between simulated middle ear injuries and their radiological appearances. Simulated ossicular chain injuries correlate well with their radiological images, and post-processing techniques provide optimal visualization of the sheep ossicles.

Background

The subtle ossicular trauma may be difficult to assess due to the small size of the structures. The precise radiological and clinical correlations of the ossicular injuries are not well documented.

Methods

The most common traumatic ossicular chain injuries were systematically simulated in the sheep temporal bone model. The images of the temporal bones were obtained with a high-resolution computed tomography scanner. The values of the dislocations were measured from the obtained images as well as in the temporal bones using calipers. Two observers independently evaluated the fine structures of the auditory ossicles using oblique multiplanar reconstructions (MPRs) and maximum intensity projections (MIPs). All segments of the facial nerve were also visualized.

Results

Optimal visualization planes of the sheep’s middle ear joints have been obtained. The coincidence of simulated ossicular injuries in the specimens and MIPs was 40%. All structures of the ossicular chain were clearly distinguished except for the stapes footplate. Evaluation of the traumatic changes of the incudostapedial joint was challenging.

Conclusions

The sheep temporal bone is a suitable model for studying the correlations between pathological alterations in the ossicular chain and their radiological appearances. The post-processing MIP technique provides a more accurate and easier diagnosis of traumatic ossicular chain injuries than MPRs alone.

## Introduction

The ossicular injuries are mainly associated with longitudinal temporal bone fractures [1]. Other causes of ossicular trauma include penetrating injuries, gunshots, and ear surgery [2]. The most common types of ossicular injuries are the separations of the incudostapedial joint (ISJ) and incudomalleal joint (IMJ), and dislocations of the incus, the incudomalleal complex, as well as stapedovestibular dislocations. The subtle ossicular trauma may be difficult to assess due to the small size of the structures [3,4]. Precise radiological and surgical correlations of trauma of the middle ear structures are not well documented. Such knowledge would be useful and important for further surgical planning and the education of the residents.

Different post-processing techniques, such as sliding-thin-slab maximum intensity projections (MIPs), can be used for the delineation of the ossicles and the facial nerve [5]. Before the introduction of high-resolution computed tomography (HRCT) scanners, injuries of the ossicular chain were investigated only after the patient’s vital functions were stabilized. Now, we have an opportunity to evaluate the traumatic changes in the middle ear using the same HRCT images without additional radiation.

The sheep ear model is extensively used in otologic research due to its remarkable similarity to the human anatomy [6,7]. There is scarce data on the radiological appearance of the ossicular chain and the facial nerve of the sheep in special literature. Sheep middle ear structures are morphologically similar or slightly smaller than those in humans [7]; therefore, radiological interpretation of the subtle changes in some corresponding structures may be even more challenging.

The main purpose of the present study was to assess the feasibility of using the sheep temporal bone model to evaluate the coincidence of the simulated ossicular injuries and their radiological appearances. We also aimed to determine the optimal visualization planes for the sheep’s middle ear structures. Additionally, we studied the effectiveness of different post-processing techniques (multiplanar reconstructions (MPRs) and MIPs) for identifying the ossicles.

## Materials and methods

Two fresh heads of 12-month-old Lithuanian Black-Headed sheep were obtained from a slaughterhouse. Four temporal bones were carefully harvested and dissected under a surgical microscope using conventional otologic instruments. The postauricular approach was used to expose structures of the middle ear. In the specimens, we simulated the most common types of traumatic ossicular chain injuries: dislocations and fractures [2] (Table 1). The simulated ossicular chain injuries were documented using an otoendoscope and video system (XE50-EcoX-TFT/USB, ILO Electronic GmbH, Quickborn, Germany). The HRCT scans of the temporal bones with each simulation were obtained using a 64-slice scanner with a slice thickness of 0.5 mm (Aquilion 64, Toshiba Medical Systems Corporation, Otawara, Japan). Image reformations were conducted using the OsiriX 32-bit version of image processing software (Pixmeo SARL, Geneva, Switzerland).

**Table 1:** Types of the ossicular injuries simulated in four sheep temporal bone specimens IM, incudomalleal; IMJ, incudomalleal joint; ISJ, incudostapedial joint; LP, lenticular process

Scan	Temporal bone 1	Temporal bone 2	Temporal bone 3	Temporal bone 4
1	ISJ separation	IMJ separation	Incudal LP fracture with superior anteromedial dislocation (0.2 mm ± 0.03 mm)	-
2	Superior anterolateral IM complex dislocation (0.3 mm ± 0.03 mm)	Incus anteromedial dislocation (0.5 mm ± 0.03 mm)	Incudal LP anteroinferior dislocation (0.2 mm ± 0.03 mm)	-
3	Stapes intravestibular subluxation (0.5 mm ± 0.03 mm)	Incus posteromedial dislocation (0.6 mm ± 0.03 mm) with clockwise 90° rotation	Incudal LP anterior dislocation (0.5 mm ± 0.03 mm)	Incus inferomedial dislocation (0.7 mm ± 0.03 mm)

The values of the dislocations in millimeters were measured from the obtained HRCT imaging data as well as in the temporal bones of sheep using calipers. Two observers, an experienced radiologist (AB) and an otosurgeon (IA), were blinded to the simulation type. The following structures were reviewed: the handle of the malleus, the head of the malleus, IMJ, the body of the incus, the long process of the incus, the short process of the incus, ISJ, the lenticular process (LP) of the incus, the stapes superstructures, and the footplate. The facial nerve and semicircular canals were delineated in their own planes as well. Each of the four specimens was reviewed by the observers with respect to the ease of distinguishing the structures. Each observer evaluated intact ossicles and ossicular chain injuries by a score from 1 to 3 (1 is indeterminable, 2 is uncertainly determinable, and 3 is obviously determinable). The image data of the intact ossicles were evaluated by each observer twice using conventional MPR and MIP techniques (slab thickness of 1.88 mm). Cohen’s correlation value was calculated to determine how well the two observers agreed on each image with simulated ossicular injuries.

No animal ethics committee approval was required for this study. All animal materials were collected postmortem from the slaughterhouse.

## Results

**Figure 1:**
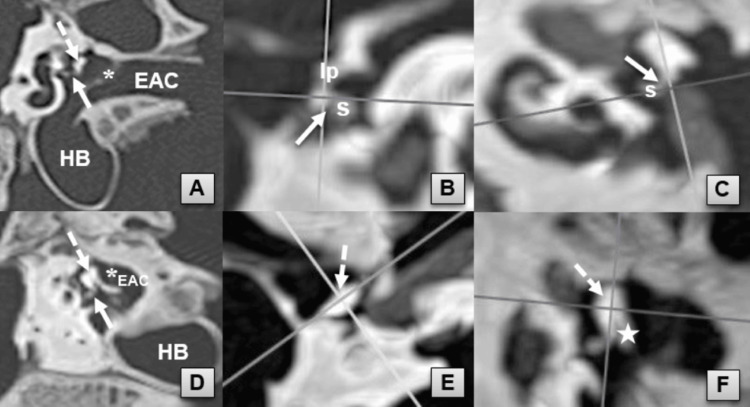
Multiplanar reformat images of ISJ and IMJ Optimal oblique coronal (A) and sagittal (D) reformat images of ISJ and IMJ, respectively. Axial (B) and sagittal (C) reconstruction reference images demonstrate the MPR planes (lines) required to view the coronal axis of ISJ. Axial (E) and coronal (F) reconstruction reference images demonstrate MPR planes (lines) required to view the sagittal axis of IMJ. EAC, external auditory canal; HB, hypotympanic bulla; IMJ, incudomalleal joint (dashed arrow); ISJ, incudostapedial joint (solid arrow); lp, long process of incus; manubrium of malleus (star); MPR, multiplanar reconstruction; s, stapes; tympanic membrane (asterisk)

**Figure 2:**
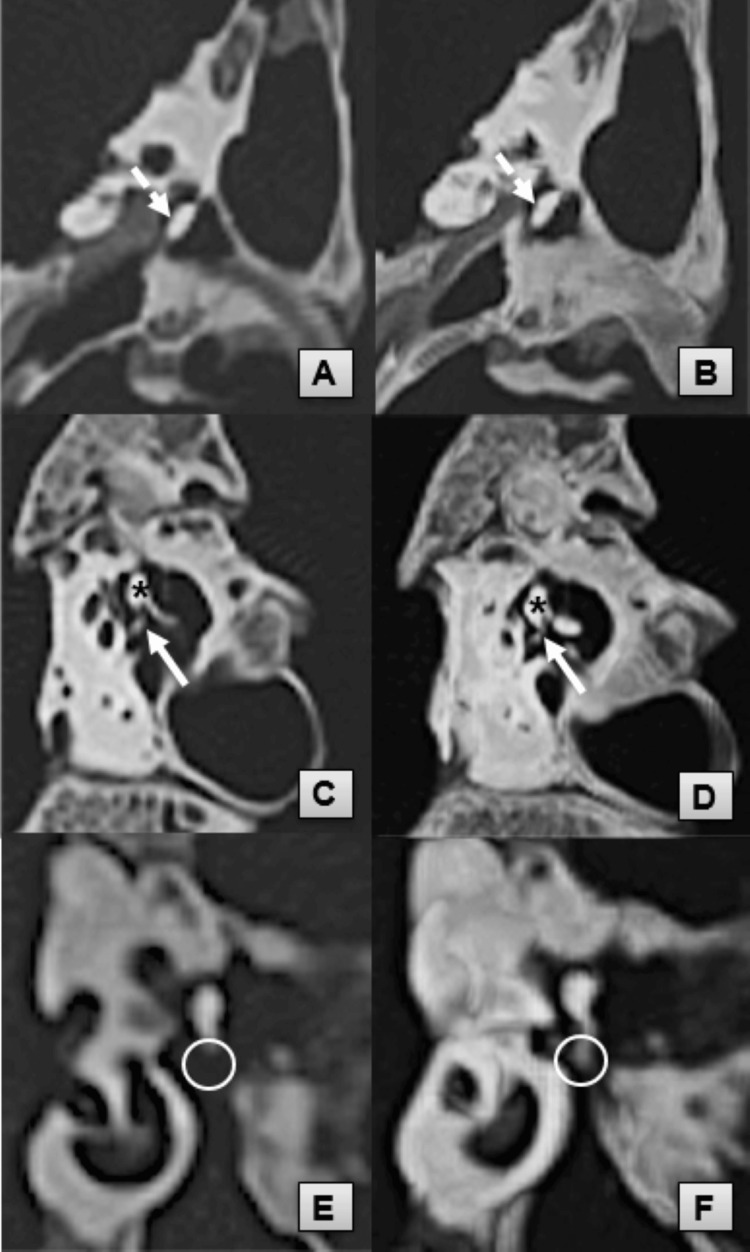
Comparative visualization of ossicular anatomy using MPR and MIP imaging techniques Subtle ossicular anatomic structures (IMJ, incudal LP, ISJ, and stapes superstructures) are clearly visualized with the MIP technique. Plain MPR images (A, C, E) in comparison with the images obtained with the MIP technique (B, D, F). IMJ, incudomalleal joint (dashed arrow); ISJ, incudostapedial joint (solid arrow); incudomalleal complex (asterisk); LP, lenticular process (circle); MIP, maximum intensity projection; MPR, multiplanar reconstruction

All macroscopically defined parts of the ossicles, except for the stapes footplate, were clearly delineated by HRCT using MPR and MIP techniques. The optimal imaging plane for the ISJ was the oblique coronal, whereas for the IMJ, it was the oblique sagittal (Figure 1). MIP reformations of the middle ear joints, as well as those of stapes superstructures, were superior to MPR. Statistically, there was a poor correlation for the otosurgeon (κ = 0.15) and a moderate correlation for the radiologist (κ = 0.41) (Figure 2).

**Figure 3:**
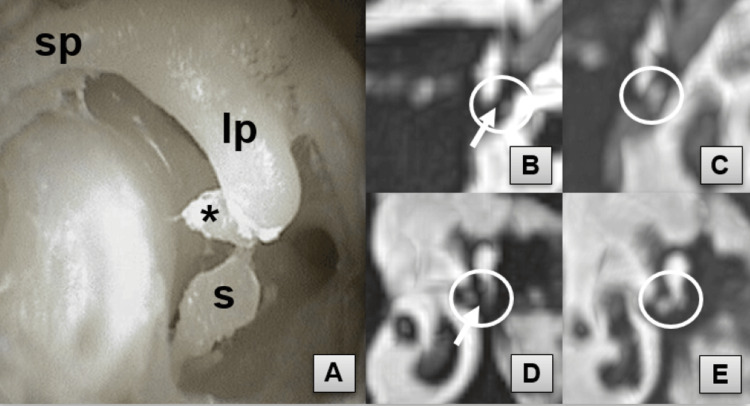
Intravestibular stapes subluxation and ISJ cleft visualization Clinical simulation, endoscopic view (A). The observers detected a widening of the ISJ cleft (arrow) on the oblique axial (B) and coronal (D) reformats in comparison with the intact ossicular chain (C, E). ISJ, incudostapedial joint (circle); lp, long process of incus; s, stapes; lenticular process (asterisk); sp, short process of incus

The overall coincidence of simulated ossicular injuries in the specimens and in MIP radiological images was 40%. This means that both observers determined a minor portion of cases of ossicular chain injuries. Evaluation of the IMJ and ISJ subtle separations (when the joints’ capsules were only separated) was challenging (Table 2). The observers experienced similar difficulties in distinguishing the incudal LP fracture with a minor superior and anteromedial dislocation of the fractured part. Only the otosurgeon identified the latter lesion. The observers quite easily determined antero-, postero-, and inferomedial dislocations of the incus. Only the experienced radiologist clearly identified the superior anterolateral incudomalleolar complex dislocation by the decreased distance between the tegmen tympani and the IMJ. The dislocations of the LP were recognized by an increased gap between the incus and stapes heads. Intravestibular stapes subluxation was detected by widening of the ISJ cleft on the oblique axial and coronal reformats (Figure 3). Cohen’s coefficient showed that there was moderate agreement between the surgeon and the radiologist (κ = 0.50).

**Table 2:** An evaluation of visualization of the ossicular injuries by an experienced radiologist and otosurgeon 1 = indeterminate; 2 = uncertainly determinable; 3 = obviously determinable ER, experienced radiologist; IM-complex, incudomalleal complex; IMJ, incudomalleal joint; ISJ, incudostapedial joint; LP, lenticular process; MIP, maximum intensity projection; MPR, multiplanar reconstruction; OS, otosurgeon

Nr	Type of injury	OS	OS	ER	ER
		MPR	MIP	MPR	MIP
1	Incus inferomedial dislocation	3	3	3	3
2	Incudal LP fracture with anteroinferior dislocation	3	3	3	3
3	Stapes intravestibular subluxation	2	3	2	3
4	Incus posteromedial dislocation	3	3	3	2
5	Incus anteromedial dislocation	1	3	3	3
6	Incudal LP fracture with anterior dislocation	1	2	2	2
7	Superior anterolateral IM-complex dislocation	1	3	1	2
8	ISJ separation	1	2	1	2
9	Incudal LP fracture with superior anteromedial dislocation	1	3	1	2
10	IMJ separation	1	1	1	1

## Discussion

**Figure 4:**
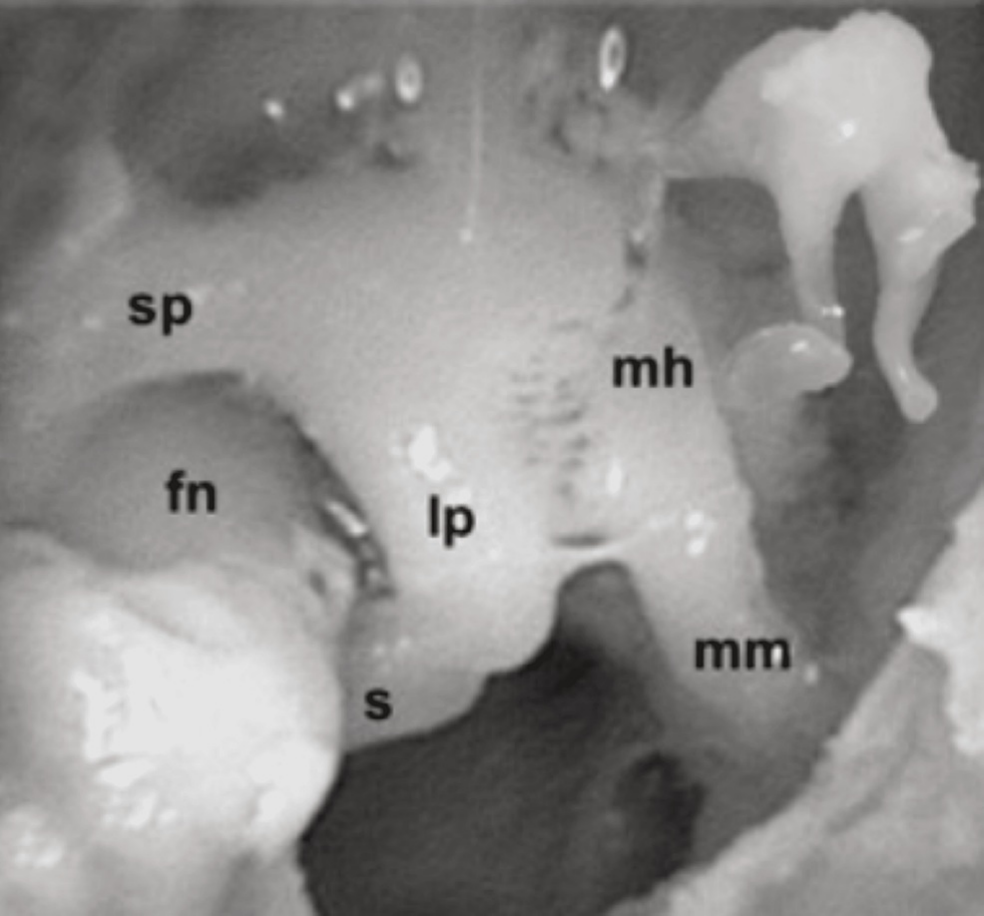
Ossicular chain of the sheep in comparison to the human ossicles fn, facial nerve; lp, long process of incus; mh, head of malleus; mm, manubrium of malleus; s, stapes; sp, short process of incus

The sheep was chosen as an animal model due to the minor differences between its ossicular chains and those of humans (Table 3). Another reason for choosing the sheep as the experimental animal is the large amount of existing data on the otic region anatomy of the sheep in the specialized literature (Figure 4) [6,7]. However, previously, some controversial data on the anatomical description of the sheep’s middle ear was reported. Lavinsky, in an anatomical and histologic study, proposed that “the articulation between the incus and stapes was similar to that found in humans but without a lenticular process” [8]. Our dissection data, similar to the results of Cordero et al. [6], clearly showed an LP (Figure 3A) articulating with the head of the stapes. More histological data is needed to clarify the anatomy and histology of the distal part of the incus in sheep.

**Table 3:** Dimensions of the sheep’s middle ear structures and differences between sheep and human temporal bone structures

Temporal bone structures		Sheep	Human
Ossicles	Malleus	Flat concave head [6]; length of malleus: 8.3 mm [8]	Concave head; length of malleus: 8-9 mm [9,10]
	Stapes	Height of stapes: 2.2 mm [8]	Height of stapes: 3 mm [9,10]
	Long process of the incus	Shorter (length: 2.8 mm) and thicker, closer to the corpus of the malleus [7,8]. The short and long processes of the incus are very similar in size [6]	Longer (length: 3.2 mm) [11] and thinner
	Manubrium of the malleus	Length: 4.9 mm [8]; thinner	Length: 4.91 mm [12]; thicker
Pars flaccida		Approximately two-thirds of the entire tympanic membrane [6]	Much smaller than pars tensa
Malleolar folds		Absent [6]	Separates pars flaccida from pars tensa
Posterior incudal ligament		Only lateral bundle [13]	Medial and lateral bundles
Mastoid		Non-pneumatized; filled with adipose tissue [6,7]	Pneumatized
Hypotympanum		Particularly large, anterior inferior position, a very wide opening of the Eustachian tube; located under the external ear canal [5]	Of various sizes. Generally smaller than mesotypanum
Facial nerve		The tympanic segment is thicker, its position is slightly more lateral, and it is usually dehiscent [6]	Tympanic segment thinner; incidence of the tympanic dehiscences: 57-74% [14,15]
Antrum		Cannot be identified [16]	Of various sizes

The results of the present study showed that the sheep temporal bone is a suitable model for the radiological study of the ossicular lesions. Even novice surgeon could easily simulate the ossicular dislocations. The simulations could easily be compared with their radiological appearance.

The sheep temporal bone model would also be useful for surgical training. Recently, a sheep model was proposed as a training model for stapedectomy [6]. The approach to the sheep ossicles was technically easy, even for the inexperienced surgeon in the present research. The dissection was carried out for 20 minutes. Therefore, novice surgeons could practice different ossiculoplasty techniques. Moreover, Lavinsky, in his excellent review on experimental and surgical otological training using sheep as a model, concluded that the following procedures could be routinely practiced: an exploratory tympanotomy, tympanoplasty, cochlea implant and implantable hearing device surgery, transcanal and chemical labyrinthectomy, and even wider experimental procedures [8].

Manipulations on the sheep incus were confirmed to be similar to the human exceptional frangibility of the LP. Yeakley had previously proposed that the fractures of the latter are difficult to identify unless they are displaced [17]. A fracture of the incudal LP with superior anteromedial dislocation was the least obvious for the observers in the present study.

We compared the radiological images to the simulations in the specimens (“the gold standard”). High interobserver agreement was obtained in the evaluation of most simulations, namely for the inferomedial, posteromedial, and anteromedial incus dislocations, the fracture of the LP with anteroinferior dislocation, and the stapes intravestibular subluxation. The subtle joint separations without ossicle dislocations were especially difficult to diagnose. Even if the joint capsule was broken by the surgical instrument, the ossicles could maintain their positions with undetectable radiological displacement.

The results of the study showed that the additional MIP technique improved the diagnostic accuracy of the visualization of the middle ear joints and their traumatic changes. We determined that MIPs were especially valuable for such subtle structures as stape superstructures. For larger structures, the technique was less important.

Furthermore, the obtained radiological data on the intact and injured sheep ossicular chains could be used for future in vivo sheep experiments [7]. While it is difficult to obtain a human temporal bone for acquiring adequate surgical skills in most otosurgical residency programs worldwide, the sheep temporal bone could be a proper model for the training [7].

A disadvantage of the study was that we applied forces directly to the ossicles, similar to the cases of penetrating or surgical iatrogenic injuries. In the case of temporal bone trauma, the origin of the ossicle injury is a transient deformation of the petrous bone with a lowering of the tegmen tympani [9].

## Conclusions

The sheep temporal bone is a suitable model for the radiological study of ossicular dislocations and surgical training. MIP images provide a more accurate diagnosis of traumatic ossicular injuries in the sheep model. The obtained radiological data on the intact and injured ossicular chains could be used for future in vivo sheep experiments.
